# Virulence analysis and antibiotic resistance of *Klebsiella pneumoniae* isolates from hospitalised patients in Poland

**DOI:** 10.1038/s41598-023-31086-w

**Published:** 2023-03-17

**Authors:** Barbara Kot, Małgorzata Piechota, Piotr Szweda, Joanna Mitrus, Jolanta Wicha, Agata Grużewska, Małgorzata Witeska

**Affiliations:** 1 Institute of Biological Sciences, Faculty of Exact and Natural Sciences, Siedlce University of Natural Sciences and Humanities, 14 Bolesława Prusa Str., 08-110 Siedlce, Poland; 2 Department of Pharmaceutical Technology and Biochemistry, Faculty of Chemistry, Gdańsk University of Technology, 11/12 G. Narutowicza Str., 80-233 Gdańsk, Poland; 3 Medical Microbiological Laboratory, Our Lady of Perpetual Help Hospital, 1/3 Gdyńska Str., 05-200 Wołomin, Poland; 4 Institute of Agriculture and Horticulture, Faculty of Agrobioengineering and Animal Husbandry, Siedlce University of Natural Sciences and Humanities, 12 Bolesława Prusa Str., 08-110 Siedlce, Poland; 5 Department of Ichthyology and Biotechnology in Aquaculture, Institute of Animal Science, Warsaw University of Life Sciences, Ciszewskiego 8, 02-786 Warsaw, Poland

**Keywords:** Microbiology, Molecular biology

## Abstract

*Klebsiella pneumoniae* (KP) is a nosocomial pathogen causing difficult-to-treat infections. The presence of virulence genes and antibiotic resistance of 109 KP isolates from hospitalized patients were investigated. Among them, 68.8% were multi-drug resistant (MDR) and 59.6% produced extended‐spectrum beta‐lactamases (ESBLs). Metallo-β-lactamases (MBLs) were produced by 22% of isolates (mainly from anus), including 16.5% of isolates producing New Delhi metallo-β-lactamase (NDM-1). The genes encoding adhesins (*fimH*—91.7%, *mrkD*—96.3%), enterobactin (*entB*—100%) and yersiniabactin (*irp-1*—88%) were frequently identified. The genes encoding salmochelin (*iroD*—9.2%, *iroN*—7.3%) and colibactin (*clbA*, *clbB*—0.9%) were identified rarely. Iron acquisition system-related *kfu* gene and *wcaG* gene involved in capsule production were identified in 6.4% and 11% of isolates, respectively. The *rmpA* gene associated with hypermucoviscosity was present in 6.4% of isolates. In 19.2% of isolates *magA* gene was detected, specific for K1 capsule serotype, while 22.9% of isolates showed K2 capsule serotype. The *rmpA*, *iroD* or *iroN* genes being diagnostic biomarkers for hypervirulent KP (hvKP) were detected in 16.5% of isolates. We found that 55.5% of hvKP were MDR and produced ESBLs, thus hospital KP isolates pose a serious threat to the healthcare system.

## Introduction

*Klebsiella pneumoniae* (KP) is an important pathogen responsible for various infections, the most frequent of which are: pneumonia, sepsis, bloodstream infections, meningitis, pyogenic liver abscesses, infections of urinary tract (UTIs) and wounds^1,2^. Among the Gram-negative bacteria, KP belongs to the major causative agents of bacteremia with a high mortality rate^3^. Infections caused by this microorganism affect mostly immunocompromised individuals. A variety of virulence factors of KP may contribute to the persistence and virulence of this microorganism. The observations indicate that KP genomes and phenotypes can change rapidly. Among KP there are the classical strains (cKP) that cause serious infections (pneumonia, UTIs, bacteremia, meningitis) in the immunocompromised individuals, including humans suffering from diabetes or malignancies^4^. Another group are hypervirulent KP strains (hvKP). They have the ability to infect both healthy and immunocompromised individuals and infections tend to be invasive; i.e., these strains can establish infection in the liver^5^, although they can also cause pneumonias, lung abscesses, and other types of infections^6^. Their high virulence correlates with the acquisition of a 200- to 220-kb plasmid containing the genes that enhance capsule production and encode siderophores^7^. The cKP strains typically cause serious nosocomial infections or UTIs and can be found worldwide, while hvKP strains are far more likely to cause community-acquired and systemic infections in otherwise healthy individuals^8,9^. The virulence and pathogenicity of KP strains are caused by a number of virulence factors including capsules, adhesins, endotoxins and iron-scavenging systems^1,10–12^. KP is an increasing threat to public health and one of the most dangerous pathogens involved in life-threatening infections, also due to the increasing resistance to antibiotics. The studies of antimicrobial resistance of KP strains revealed their widespread resistance to aminoglycosides, fluoroquinolones, cephalosporins and carbapenems^12^. KP developed several mechanisms to evade the activity of antibiotics, including the ability to produce extended‐spectrum beta‐lactamases (ESBLs) and various carbapenemases. During the past decades, KP has been considered a dangerous infectious agent causing an increasing number of severe infections (pneumonias, bloodstream infections), and showing increasing scarcity of effective treatments. It is the result of additional genetic traits acquired by cKP strains that led to the emergence of hvKP or antibiotic resistant strains. Taking into consideration increasing threat caused by this microorganism to public health worldwide, we undertook the present study the aim of which was to examine the presence of important virulence genes encoding fimbriae, siderophores, capsules and hypercapsule in 109 KP isolates from various clinical materials and to determine genetic virulence profiles specific for KP isolates causing various infections in hospitalized patients in a district hospital in central Poland. We also determined susceptibility of these isolates to selected antibiotics and assessed the prevalence of hypervirulent and multi-drug resistant (MDR) isolates which is important for establishing of treatment strategy of infections caused by this microorganism.

## Results

### Resistance to antibiotics

**Figure 1:**
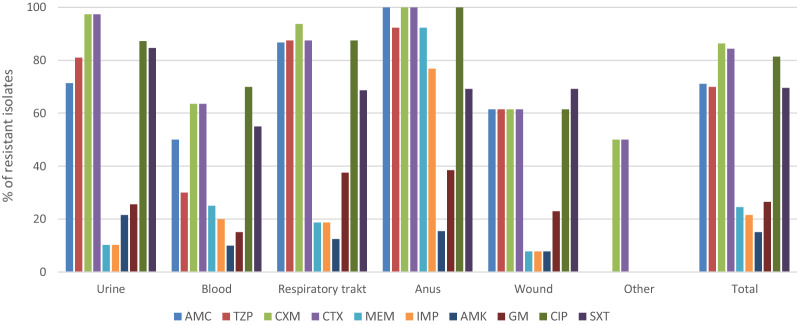
Antimicrobial resistance of *K. pneumoniae* isolates from different clinical materials. AMC—amoxicillin plus clavulanic acid, TZP—piperacillin plus tazobactam, CXM—cefuroxime, CTX—cefotaxime, MEM—meropenem, IMP—imipenem, AMK—amikacin, GM—gentamicin, CIP—ciprofloxacin, SXT—trimethoprim plus sulfamethoxazole.

High percentage of KP isolates from human clinical materials were resistant to penicillins combined with β-lactamase inhibitors (amoxicillin plus clavulanic acid—71.1% and piperacillin plus tazobactam—70.0%) (Fig. 1). Significantly more isolates (100%) from anus were resistant to amoxicillin plus clavulanic acid than from urine (71.4%) (*p* = 0.0305), wound (61.5%) (*p* = 0.0155) and blood (50%) (*p* = 0.0244). Resistance to this antibiotic was also more frequent in isolates from respiratory tract (86.7%) than in isolates from blood (*p* = 0.0026).

Resistance of isolates from blood to piperacillin plus tazobactam was lower (30%) compared to those from anus (92.3%, *p* = 0.0006), respiratory tract (87.5%, *p* = 0.0006) and urine (81.0%, *p* = 0.0002).

High percentage of KP isolates showed resistance to the second (cefuroxime, 86.4%) and third (cefotaxime, 84.4%) generation cephalosporins. All isolates from anus and 97.4% of isolates from urine showed resistance to these antibiotics. Significantly more isolates from urine were resistant to cefuroxime and cefotaxime compared to the isolates from blood (63.6%, *p* = 0.0002 and 63.6%, *p* = 0.0006, respectively) and wound (61.5%, *p* = 0.0004 and 61.5%, *p* = 0.0008, respectively). Among the isolates resistant to carbapenems (meropenem—24.5% and imipenem—21.5%), the isolates from anus were the most frequent (Fig. 1). The percentage of isolates from anus resistant to meropenem (92.3%) was higher than the isolates from urine (10.2%) and wound (7.7%) (*p* < 0.0001), respiratory tract (18.7%) (*p* = 0.0001) and blood (25%) (*p* = 0.0002). Resistance of isolates from anus to imipenem (76.9%) was also higher compared to the isolates from urine (10.2%) (*p* < 0.0001), respiratory tract (18.7%) (*p* = 0.0022), blood (20%) (*p* = 0.0014) and wound (7.7%) (*p* = 0.0005). The percentage of KP isolates resistant to amikacin and gentamicin was 15% and 26.5%, respectively. The investigated KP isolates showed high resistance to ciprofloxacin (81.4%). All isolates from anus were resistant to this antibiotic. High percentage of resistant isolates was obtained from urine (87.2%) and respiratory tract (above 87.5%). About 70% of isolates were resistant to trimethoprim plus sulfamethoxazole. The percentage of isolates resistant to this chemotherapeutic was significantly higher among the urine isolates (84.6%) compared to the isolates from wounds (53.8%, *p* = 0.0499). ESBLs were produced by 59.6% of isolates (Table 1). The highest percentage of ESBL producers was detected among isolates from urine (87.1%) and respiratory tract (68.7%). All K54 isolates and 76.2% of K1 isolates showed ESBL production (Table 2). MBLs were detected in 22% of isolates, including 16.5% of isolates producing NDM-1 (Table 1). Percentage of isolates from anus that produced NDM-1 (58.8%) was significantly higher compared to the isolates from other clinical materials. The isolates with NDM-1 belonged to K2 (28%) and K57 (25%) serotypes (Table 2). Carbapenemase class A (KPC) was detected only in two isolates. In the investigated isolates no OXA-48 carbapenemase was detected.

**Table 1:** The prevalence of resistance mechanisms in *K. pneumoniae* isolated from different clinical materials.

Source of isolation (n)	Resistance mechanisms, n (%)				
	ESBLs	KPC	MBLs	NDM-1	OXA-48
Urine (39)	34 (87.1)	0	3 (7.7)	1 (2.5)	0
Blood (22)	9 (40.9)	1 (4.5)	5 (22.7)	4 (18.2)	0
Respiratory tract (16)	11 (68.7)	0	3 (18.7)	2 (12.5)	0
Anus (17)	3 (17.6)	1 (5.8)	12 (70.5)	10 (58.8)	0
Wound (13)	7 (53.8)	0	1 (7.7)	1 (7.7)	0
Other (2)	1 (50)	0	0	0	0
Total (109)	65 (59.6)	2 (1.8)	24 (22.0)	18 (16.5)	0

ESBLs—extended spectrum beta-lactamases, KPC—carbapenemase class A, MBLs—metallo-β-lactamases, NDM-1—New Dehli metallo-β-lactamase, OXA-48—carbapenemase class D.

**Table 2:** The prevalence of resistance mechanisms in *K. pneumoniae* belonging to different K serotypes.

K serotype (n)	Resistance mechanisms, n (%)			
	ESBLs	KPC	MBLs	NDM-1
K1 (21)	16 (76.2)	0	0	0
K2 (25)	10 (47.6)	1 (4.0)	7 (28.0)	7 (28)
K54 (4)	4 (100)	0	0	0
K57 (40)	21 (52.5)	1 (2.5)	14 (35.0)	10 (25)
Other (19)	14 (73.7)	0	3 (15.8)	1 (5.2)

ESBLs—extended spectrum beta-lactamases, KPC—carbapenemase class A, MBLs—metallo-β-lactamases, NDM-1—New Dehli metallo-β-lactamase, OXA-48—carbapenemase class D.

Out of the investigated isolates, 75 (68.8%) were MDR, 30 (40%) of which showed resistance to 4 groups of antibiotics, while remaining were resistant to 3 groups. The highest percentage of MDR isolates was found among those from respiratory tract (93.7%) urine (79.5%) and anus (64.7%) (Table 3). Among K1 and K2 isolates, 52.4% and 64% isolates, respectively, were MDR.

**Table 3:** Multi-drug resistance pattern of *K. pneumoniae* isolates from different clinical materials.

Combination of drugs (No. of antimicrobial agents classes)	No. of isolates	Resistance mechanisms (n)	Source (n)	Hospital wards (n)
AMC,TZP,CTX,CXM,SXT,CIP (3)	21	ESBLs (21)	Urine (11), respiratory tract (5), wound (4), blood	Nephrology (3), Intensive Care (4), Neurological (2), Internal (8), Orthopaedics, Surgery (3)
TZP,CTX,CXM,SXT,CIP (3)	5	ESBLs (5)	Urine (4), respiratory tract	Neurological, Internal (2), Nephrology (2)
AMC,CTX,CXM,SXT,CIP (3)	4	ESBLs (4)	Urine (2), blood (2)	Internal (3), Neurological
CTX,CXM,SXT,CIP (3)	3	ESBLs (3)	Blood (2), urine	Nephrology, Internal (2)
AMC,TZP,CTX,CXM,GM, AMK, CIP (3)	1	ESBLs (1)	Urine	Nephrology
AMC,TZP,CTX,CXM,GM,SXT,CIP (4)	10	ESBLs (10)	Wound (2), respiratory tract (5), urine (2), blood	Surgery (4), Internal, Nephrology (2), Internal (2), Neurological
AMC,TZP,CTX,CXM,AMK,SXT,CIP (4)	4	ESBLs (4)	Urine (3), respiratory tract	Nephrology, Dialysis station, Intensive Care, Internal
AMC,CTX,CXM,AMK, SXT,CIP (4)	1	ESBLs (1)	Anus	Neurological
AMC,CTX,CXM,GM, AMK, SXT,CIP (4)	1	ESBLs (1)	Blood	Internal
AMC,CTX,CXM,GM,SXT,CIP (4)	1	ESBLs (1)	Urine	Intensive Care
AMC,TZP,CTX,CXM,GM,AMK,SXT,CIP (4)	1	ESBLs (1)	Wound	Surgery
TZP,CTX,CXM,GM,SXT,CIP (4)	3	ESBLs (3)	Urine (3)	Nephrology
AMC,TZP,CTX,CXM,MEM,IMP,GM,AMK,SXT,CIP (4)	1	MBL (1)	Urine	Nephrology
AMC,TZP,CTX,CXM,MEM,IMP,GM,SXT,CIP (4)	6	NDM-1 (6)	Anus (4), urine, blood	Internal (2), Intensive Care (3), Surgery
AMC,TZP,CTX,CXM,MEM,IMP,SXT,CIP (3)	6	NDM-1 (6)	Anus (4), blood (2)	Internal (2), Intensive Care (2), Surgery (2)
AMC,TZP,CTX,CXM,MEM,IMP,AMK,SXT,CIP (4)	2	MBLs (2)	Blood, urine	Internal (2)
AMC,TZP,CTX,CXM,MEM,IMP,GM,CIP (3)	3	NDM-1 (3)	Respiratory tract (2), anus	Neurological (2), Surgery
AMC,TZP,CTX,CXM,MEM,IMP,AMK,CIP (3)	2	MBLs (2)	Respiratory tract, anus	Neurological, Intensive Care

AMC—amoxicillin plus clavulanic acid, TZP—piperacillin plus tazobactam CTX—cefotaxime, CXM—cefuroxime, MEM—meropenem, IMP—imipenem, AMK—amikacin, GM—gentamicin, CIP—ciprofloxacin, SXT—trimethoprim plus sulfamethoxazole. ESBLs—extended spectrum beta-lactamases, MBLs—metallo-β-lactamases, NDM-1—New Dehli metallo-β-lactamase.

### Virulence-associated genes in *K. pneumoniae* isolates

Analysis of the frequency of genes encoding virulence factors in 109 KP isolates showed that the genes encoding adhesins were commonly found (Table 4). The *fimH* gene encoding the minor subunit FimH placed on the tip of type 1 fimbriae was present in 91.7% of isolates, while the presence of *mrkD* gene encoding adhesin MrkD located at the tip of type 3 fimbriae was detected in 96.3% of isolates. Among the genes encoding siderophores, *entB* gene encoding enterobactin was identified in all isolates. The *irp-1* gene encoding yersiniabactin was also commonly identified (88%), although less frequently in isolates from wound (69.2%) compared to the urine (92.3%, *p* = 0.0389) and anus isolates (100%, *p* = 0.0153). Genes encoding other siderophores such as salmochelin (*iroD*—9.2% and *iroN*—7.3%) were identified rarely. The *iroD* gene was more frequent among isolates from wounds (23%) than from blood and anus (*p* = 0.0405). The highest prevalence of *iroN* gene was observed in isolates from the respiratory tract (18.7%) and it was higher than in isolates from urine (2.6%, *p* = 0.0378). Iron acquisition system-related *kfu* gene and *wcaG* gene involved in capsule production were identified in 6.4% and 11% of isolates, respectively, and the occurrence of these genes in isolates from different clinical specimens did not differ significantly. Genes encoding colibactin (*clbA* and *clbB*) were present only in 0.9% of isolates. The *rmpA* gene associated with the hypercapsule (hypermucoviscous phenotype) was present in 7 (6.4%) isolates obtained from urine (2), blood (3) and wounds (2). The mucoviscosity-associated gene A (*magA*) specific to K1 capsule serotype was detected in 19.2% of isolates. The prevalence of this gene did not differ significantly among the isolates from various clinical specimens and it was absent from the rectal swab isolates. Higher percentage of isolates belonged to K2 capsule serotype (22.9%). The K2 serotype was more frequent among the isolates from the respiratory tract (50%) compared to those obtained from urine (15.4%, *p* = 0.008) and anus (11.7%, *p* = 0.0188). The remaining isolates from anus (88.3%) belonged to K57 serotype and were more frequent compared to K57 isolates from the respiratory tract and blood (22.7%, *p* < 0.0001), urine (33.3%, *p* = 0.0002) and wounds (30.7%, *p* = 0.0016). The serotypes K5 and K20 were not detected in the investigated KP isolates, while 3.7% of isolates belonged to K54 type.

**Table 4:** The prevalence of virulence-associated genes in *K. pneumoniae* isolates from different clinical materials.

Genes	Source of isolation						
	Urine n = 39 (%)	Blood n = 22 (%)	Respiratory tract n = 16 (%)	Anus n = 17 (%)	Wound n = 13 (%)	Other n = 2 (%)	Total n = 109 (%)
Adhesins							
*fimH*	35 (89.7)	19 (86.4)	15 (93.7)	17 (100)	12 (92.3)	2 (100)	100 (91.7)
*mrkD*	35 (89.7)	22 (100)	16 (100)	17 (100)	13 (100)	2 (100)	105 (96.3)
Salmochelin							
*iroD*	4 (10.2)	0	3 (18.7)	0	3 (23.0)	0	10 (9.2)
*iroN*	1 (2.6)	2. (9.1)	3 (18.7)	0	2 (15.4)	0	8 (7.3)
Colibactin							
*clbA*	1 (2.6)	0	0	0	0	0	1 (0.9)
*clbB*	1 (2.6)	0	0	0	0	0	1 (0.9)
Enterobactin							
*entB*	39 (100)	22 (100)	16 (100)	17 (100)	13 (100)	2 (100)	109 (100)
Yersiniabactin							
*irp-1*	36 (92.3)	19 (86.3)	14 (87.5)	17 (100)	9 (69.2)	1 (50)	96 (88.0)
Iron uptake system							
*kfu*	5 (12.8)	1 (4.5)	1 (6.25)	0	0	0	7 (6.4)
Capsular fucose synthesis							
*wcaG*	5 (12.8)	3 (13.6)	1 (6.2)	1 (5.9)	2 (15.4)	0	12 (11.0)
Regulator of mucoid phenotype A							
*rmpA*	2 (5.1)	3 (13.6)	0	0	2 (15.4)	0	7 (6.4)
Capsular types (K)							
K1	9 (23.0)	4 (18.2)	4 (25.0)	0	3 (23.0)	1 (50)	21 (19.2)
K2	6 (15.4)	6 (27.3)	8 (50.0)	2 (11.7)	3 (23.0)	0	25 (22.9)
K5	0	0	0	0	0	0	0
K20	0	0	0	0	0	0	0
K54	2 (3.7)	0	0	0	2 (9.0)	0	4 (3.7)
K57	13 (33.3)	5 (22.7)	2 (12.5)	15 (88.3)	4 (30.7)	1 (50)	40 (36.7)

The *rmpA,* a hypercapsule-associated gene, was detected in one K1 isolate and in three K2 and K54 isolates (Table 5). In all isolates with *rmpA*, *entB* and *irp-1* siderophore genes were detected, and some isolates with *rmpA* gene also had other siderophore genes such as *iroD*, *iroN,* and genes encoding colibactin (*clbA* and *clbB*). In 19% of K1 isolates *kfu* and *wcaG* genes were present. The *wcaG* gene was also found in K2 (16%), K57 (5%) and K54 (50%) isolates. The most common virulotype: *entB*, *irp-1*, *fimH*, *mrkD* occurred in isolates belonging to different K serotypes. Among the investigated KP isolates, in 18 (16.5%) *rmpA*, *iroD* or *iroN* genes were detected, being diagnostic biomarkers for hvKP. In a group of hvKP, 10 isolates (55.5%) produced ESBLs, however, they did not produce MBL, including NDM-1, and did not produce KPC. These isolates were MDR and showed resistance to 3 or 4 classes of antibiotics (Table 6).

**Table 5:** Distribution of virulence-associated genes among K serotypes of *K. pneumoniae.*

K serotype (n)	Regulator of mucoid phenotype	Siderophore genes				Colibactin		Iron uptake system	Capsular fucose synthesis	Adhesins genes		No. of isolates
	*rmpA*	*iroD*	*iroN*	*entB*	*irp-1*	*clbA*	*clbB*	*kfu*	*wcaG*	*fimH*	*mrkD*	
K1 (21)	+	−	−	+	+	−	−	−	−	+	+	1
	−	−	−	+	+	−	−	+	−	+	+	2
	−	−	−	+	+	−	−	+	+	+	+	1
	−	−	−	+	+	−	−	−	−	+	+	7
	−	+	+	+	+	−	−	−	−	+	+	1
	−	+	−	+	+	−	−	−	−	+	+	1
	−	−	−	+	+	−	−	−	+	+	+	2
	−	−	−	+	+	−	−	−	−	+	+	2
	−	+	−	+	−	−	−	−	+	+	+	1
	−	−	−	+	−	−	−	+	−	−	+	1
	−	−	−	+	−	−	−	−	−	+	+	2
K2 (25)	+	+	+	+	+	−	−	−	−	+	+	2
	+	−	−	+	+	−	−	−	−	+	+	1
	−	+	+	+	−	−	−	−	−	+	+	1
	−	+	+	+	+	−	−	−	+	+	+	2
	−	+	−	+	+	−		−	−	+	+	1
	−	−	−	+	+	−	−	−	−	+	+	12
	−	−	−	+	+	−	−	−	+	+	+	1
	−	−	−	+	+	−	−	−	−	+	−	1
	−	−	−	+	−	−	−	−	−	+	+	3
	−	−	−	+	−	−	−	−	+	+	+	1
K57 (40)	−	−	−	+	+	−	−	−	−	+	+	35
	−	−	−	+	−	−	−	−	−	+	+	2
	−	−	−	+	+	−	−	−	+	+	+	2
	−	−	−	+	+	−	−	−	−	+	−	1
K54 (4)	+	−	−	+	+	−	−	−	−	+	−	1
	+	−	−	+	+	+	+	−	−	−	−	1
	+	−	−	+	+	−	−	−	+	−	+	1
	−	−	+	+	−	−	−	−	+	−	+	1
Other (19)	−	−	−	+	+	−	−	−	−	−	+	1
	−	−	+	+	+	−	−	+	−	−	+	1
	−	−	−	+	+	−	−	+	−	+	+	2
	−	−	−	+	−	−	−	−	−	−	+	1
	−	−	−	+	+	−	−	−	−	+	−	1
	−	−	−	+	+	−	−	−	−	−	+	1
	−	−	−	+	+	−	−	−	−	+	+	12

**Table 6:** Multidrug-resistance of hvKp isolates producing ESBLs.

Source	Combination of drugs (No. of antimicrobial agents classes)	Virulence genes
Blood	CTX,CXM,SXT,CIP (3)	*rmpA,entB,irp-1,mrkD*
Urine	AMC,CTX,CXM,GM,SXT,CIP (4)	*rmpA,entB,irp-1,clbA,clbB*
Urine	AMC,TZP,CTX,CXM,SXT,CIP (3)	*rmpA,entB,irp-1,mrkD,wcaG*
Urine	AMC,TZP,CTX,CXM,SXT,CIP (3)	*iroD,entB, irp-1,mrkD*
Urine	TZP,CTX,CXM,SXT,CIP (3)	*iroD,entB, irp-1,mrkD, fimH*
Urine	AMC,TZP,CTX,CXM,SXT,CIP (3)	*iroD,iroN,entB,irp-1,mrkD, fimH,wcaG*
Respiratory tract	AMC,TZP,CTX,CXM,SXT,CIP (3)	*iroD,iroN,entB, irp-1,mrkD, fimH*
Respiratory tract	AMC,CTX,CXM,SXT,CIP (3)	*iroD,iroN,entB,irp-1,mrkD, fimH,wcaG*
Blood	AMC,CTX,CXM,GM,AMK,SXT,CIP (4)	*iroN,entB, irp-1,mrkD,kfu*
Blood	AMC,CTX,CXM,TZP,GM,SXT,CIP (4)	*iroN,entB,mrkD*

AMC—amoxicillin plus clavulanic acid, TZP—piperacillin plus tazobactam CTX—cefotaxime, CXM—cefuroxime, AMK—amikacin, GM—gentamicin, CIP—ciprofloxacin, SXT—trimethoprim plus sulfamethoxazole.

## Discussion

KP is responsible for various healthcare-associated infections, such as wound, bloodstream, urinary tract and respiratory tract infections. Infections caused by KP not only increase the mortality rates in patients, but also prolong hospitalization and increase treatment costs. The recorded prevalence of KP nosocomial infections was up to 10%^13^. The extensive use of antimicrobials in treating these infections led to high incidence of resistance in KP. MDR KP was first reported in the United States, then in Europe, South America and Asia^14^. Management of antimicrobial resistance in MDR KP is a major challenge for clinicians because few effective antibiotics are available, resulting in increased mortality rates, longer hospitalization and excessive treatment costs^14^. In our study, we showed that 68.8% of isolates obtained in 2018–2022 from different clinical specimens from hospitalized patients in central Poland were MDR. The results of meta-analysis performed by Asri et al.^15^ showed that the pooled prevalence of nosocomial MDR KP was estimated at 32.8%. This analysis also showed the differences between countries and regions. The results from North America showed the lowest value of 12.9%, while the data from South America—72.4%^15^. Very high percentage of MDR KP was found in Egyptian hospitals (77.7%)^1^, Brazil (61.9%)^16^ and Bangladesh (82%)^17^. In various locations in Nepal, the frequency of MDR KP ranged from 6 to 91%^18^. In our study, 59.6% of KP isolates produced ESBLs and this resistance mechanism was present in 73.3% of MDR isolates. Similar result (above 60% isolates with ESBLs) was obtained by Sękowska et al.^19^, while other studies from Poland showed that the percentage of isolates producing ESBLs ranged from 40 to 70% depending on the region, ward/hospital, and species of bacteria^19^. In our study, the highest percentage of *K. pneumoniae* isolates producing ESBLs was found among the isolates from urine (87.1%) and respiratory tract (68.7%), while the lowest among rectal isolates (17.6%). All MDR isolates producing ESBLs were resistant to the second and third generations cephalosporins and also to ciprofloxacin. Additionally, almost all MDR KP isolates with ESBLs were resistant to penicillins combined with β-lactamase inhibitors, and 72% of them were resistant to trimethoprim plus sulfamethoxazole. These isolates were sensitive to meropenem and imipenem, additionally 77.4% and 89.4% of isolates were sensitive to gentamicin and amikacin, respectively. These results show that carbapenems and aminoglycosides should be considered as priority antibiotic therapy for infections caused by ESBL-producing KP isolates which was also suggested by Khaertynov et al.^20^ Howewer, the recent study showed that carbapenem-resistant KP represents the fastest growing antibiotic resistance threat in Europe, in terms of human morbidity and mortality. It has been shown that in 2007–2015 the number of infections and number of deaths caused by carbapenem-resistant KP in the EU increased by 6.16 times^21^. The rate of carbapenem resistance in KP in China increased from 2.9% in 2005 to 24.4% in 2021^22^. In our study, MBLs were found in 24 (22%) isolates. Lower percentage (16.9%) of nosocomial isolates producing MBLs was obtained by Sękowska et al.^19^ In our study, the production of NDM-1 was detected in 16.5% of isolates, the most frequently from anus (58.8% of isolates). Similarly, Sarowska et al.^23^ showed that among carbapenem-resistant KP isolates obtained from patients of the specialist hospital in Poland (Wrocław), isolates derived from rectal swabs were dominant and comprised 81.3%. In our study, the resistance to meropenem (92.3%) and imipenem (76.9%) among isolates from anus was significantly more frequent compared to the isolates from other clinical materials. For this reason, in order to limit the emergence of epidemic outbreaks, patients admitted to hospital wards, should be tested for the presence of carbapenem-resistant KP isolates. In the case of carbapenemase-producing isolates, the therapeutic options are very limited. The treatment of these infections requires coordinated interventions and the use of three or four drugs^24^. In our study, KP isolates producing NDM-1 comprised 20% of the MDR isolates resistant to penicillins combined with β-lactamase inhibitors, cephalosporins, ciprofloxacin and trimethoprim plus sulfamethoxazole. Some isolates also showed resistance to aminoglycosides.

We also investigated the presence of selected important virulence genes encoding fimbriae, siderophores, capsules and hypercapsule, genetic virulence profiles specific for KP isolates and the prevalence of hvKP isolates since these data seem essential to develop effective strategies for treating infections caused by these microorganisms.

Type 1 and 3 fimbriae are mediators of KP adhesion that have been characterized as important pathogenicity factors during infections^2^. Type 1 fimbriae bind D-mannosylated glycoproteins and are expressed in 90% of both clinical and environmental KP isolates^25^. In our research, the *fimH* gene encoding the minor subunit FimH responsible for adhesive properties of these fimbriae was found in 91.7% of isolates. Type 3 fimbriae with the adhesin MrkD being located at the tip bind extracellular matrix proteins such as type IV and V collagens^26^. Type 3 fimbriae were reported to be crucial for biofilm production^27^. They are expressed during biofilm formation on catheters, therefore probably may contribute to the occurrence of UTIs. They also assist KP in the colonization of endotracheal tubes and thus may promote lung infection^28^. In the current study, the *mrkD* gene was present in above 96% of isolates.

The ability to acquire iron during infection is necessary for KP pathogenesis. Iron is not readily available for bacteria in the host during infection, because, as part of the nonspecific immune response, iron is bound to the iron transport molecules such as transferrin. Iron level in mammalian hosts is also reduced by binding to lactoferrin, an innate defence protein present in body fluids^29^. The production of siderophores, molecules with higher affinity to iron than host transport proteins, is the dominant strategy used by many pathogens. KP exhibits several siderophores, including enterobactin, yersiniabactin and salmochelin.

In our study, *entB* gene encoding enterobactin was identified in all isolates which is consistent with the results obtained by other authors^30^. Enterobactin expression is ubiquitous among both classical and hvKP isolates and it is therefore considered the primary iron uptake system used by KP^3,11^.

The *irp* gene encoding yersiniabactin was also frequently identified in this study (88%), although this gene was significantly less frequently identified in wound isolates than in urine and anal isolates. Bachman et al.^31^ showed that KP isolates with *irp-1* were significantly overrepresented among respiratory tract isolates. The results of our study also showed that isolates from the respiratory tract with the *irp-1* gene were numerous but their percentage (87.5%) did not differ compared the urine, anus and blood isolates. Yersiniabactin is expressed during lung infection, and its activity, contrary to enterobactin, is not inhibited by lipocalin-2^31,32^. Lipocalin-2 is a protein released from neutrophils during infections. However, yersiniabactin is unable to acquire the iron required for the growth of KP in the presence of the host protein transferrin^31^. Thus, strains that produce yersiniabactin alone are not capable of disseminating from the lungs, because plasma transferrin prevents the growth of KP in blood. Therefore, production of multiple siderophores by KP facilitates colonization of various tissues in case when one siderophore is neutralized by the host^33^. The yersiniabactin is associated with severe diseases in humans, and is produced by both hvKP and cKP strains^34^. In our study, the isolates showing other genes encoding siderophores were also found. Genes encoding salmochelin biosynthesis (*iroD*—9.2%) and salmochelin receptor gene (*iroN*—7.3%) were rarely identified. The *iroD* gene was significantly more frequently detected among the wound than from blood and anal isolates. Whereas, *iroN* gene was the most frequently identified in the respiratory tract isolates. Salmochelin is a c-glycosylated form of enterobactin^35,36^, and this modification prevents binding and neutralization by lipocalin-2^36^. Thus, salmochelin enhances KP colonization of the nasopharynx, and salmochelin-producing strains are more virulent. Salmochelin is present in only about 2–4% of nosocomial KP strains but is much more prevalent in hvKP strains^30,34,37^.

Kfu is an iron transport system being an important virulence factor associated with hvKP strains^38,39^. The *kfu* gene is more frequently found in invasive human clinical strains, particularly in those from a liver abscess and strains causing meningitis than in noninvasive strains^40^. This indicates that iron acquisition by KP with the participation of Kfu is important for development of clinical infection. In our study, iron acquisition system-related *kfu* gene was identified in 6.4% of isolates and the occurrence of this gene in isolates from various clinical specimens did not significantly differ.

The hvKP was identifed for the first time in Taiwan in 1986 as a common cause of liver abscesses in young, healthy individuals^5,41^. The hvKP strains cause disseminated multi-site infections and express highly mucoid capsules, creating a hypermucoviscous colony phenotype. Hypermucoviscosity involves the presence of mucoviscous exopolysaccharide, more robust than that of a typical capsule. The hvKP are usually more virulent in mouse models than cKp strains^34^. High virulence is caused by the presence of one of several large hypervirulence plasmids^42,43^. One of two pathogenicity loci on these plasmids (PAL-2) usually consists of a distinct mucoid regulator operon (*rmpADC*) and genes encoding iron-acquisition factors (salmochelin and aerobactin)^34^. In this study, we showed the presence of *rmpA* gene (regulator of mucoid phenotype A) in 7 (6.4%) isolates from urine, blood and wound. The results obtained by Russo et al.^44^ revealed that the *rmpA* and *iroB* genes were laboratory markers for identifying hvKp with a high degree of accuracy. In our study, we examined the presence of *rmpA*, *iroD* and *iroN* in isolates of KP from hospitalized patients and showed that 16.5% of the isolates contained one, two or all of the genetic features of the hvKp strains. Genes encoding colibactin, which is a non-ribosomal peptide-polyketide with the ability to induce host DNA damage and promote colorectal cancer^45,46^, have also been detected in some *rmpA* isolates. Antibiotic resistance is a phenomenon associated primarily with cKP but there have been reports of hvKP strains carrying ESBLs or carbapenemases^47,48^. Kochan et al.^34^ stressed that hvKP strains remained susceptible to most antibiotics but changes in their susceptibility were observed. The number of infections caused by antimicrobial-resistant hvKP is increasing, probably due to the increase in hospital-acquired hvKp infections. The results of our study confirm this trend and showed that 55.5% of hvKP isolates were MDR and produced ESBLs. These isolates were resistant to penicillins combined with β-lactamase inhibitors, the second and third generation cephalosporins, ciprofloxacin, trimethoprim plus sulfamethoxazole and some of them to aminoglicosides. MDR hvKP may spread in the hospital environment in two ways. One—when hvKP isolate acquires an antibiotic resistance mobile element, and other—when an MDR-cKP isolate acquires a plasmid or integrative and conjugative element containing hvKP virulence genes^34^. Increasing number of KP strains that are both antibiotic-resistant and hypervirulent can pose a serious threat to the healthcare system.

In our study, the chromosomal mucoviscosity-associated gene A (*magA*) specific to K1 capsule serotype was detected in 19.2% of isolates. The prevalence of this gene did not significantly differ among the isolates from various clinical specimens, but it was absent from the rectal isolates. K1 and K2 strains are generally more virulent than strains of other serotypes^2^ because they may inhibit production of reactive oxygen species by human neutrophils and thus survive better in tissues compared to the other serotypes^49^. In addition, strains of the K1 and K2 serotypes are more resistant to phagocytosis and intracellular killing by alveolar macrophages and neutrophils than other strains because they lack specific mannose residue repeats that are recognized by the mannose receptor on macrophages^50^. In our study, the percentage of K2 isolates was higher than K1 isolates, which is consistent with the results obtained by other researchers^49^ who reported that K2 isolates were more prevalent among clinical isolates than K1. Hypercapsule encoded by *magA* can be produced in the absence of *rmpA*^6^. In our study the *rmpA* gene associated with the hypermucoviscous phenotype was detected in one K1 isolate and in three K2 and K54 isolates. In this study, we also investigated the presence of *wcaG* gene in KP isolates. The *wcaG* gene that encodes capsular fucose synthesis and enhances the ability of bacteria to evade phagocytosis^51^ was identified in 11% of isolates, and the occurrence of this gene in isolates from various clinical specimens did not significantly differ. We found that *wcaG* was present in both hvKP and cKP isolates that belonged to different serotypes which is consistent with the results obtained by other authors^52^.

## Conclusion

KP is an important nosocomial pathogen, often associated with nosocomial outbreaks, showing different degrees of virulence and sensitivity to antibiotics. The emergence of a number of difficult-to-treat infections caused by KP poses a challenge for the medical community to assess the bacterial factors critical during infection. In order to best understand this diverse pathogen, we investigated the presence of virulence genes in various isolates obtained from respiratory tract infections, UTIs, blood, wounds and digestive tract colonization. We also determined susceptibility of these isolates to antibiotics and evaluated the prevalence of hypervirulent and MDR isolates. We showed that a high percentage of nosocomial KP isolates were MDR and produced ESBLs. The isolates with MBLs, including NDM-1, were the most frequently identified among rectal isolates. Our results also showed that genes encoding adhesins (*fimH* and *mrkD*) and siderophores (*entB* and *irp-1*) were frequently identified in the investigated isolates. The *rmpA* gene associated with hypermucoviscous phenotype and siderophore genes encoding salmochelin that are diagnostic biomarkers for hvKP were also detected. Among hvKP, above 50% were MDR and produced ESBLs. Increasing numbers of KP isolates that are both antibiotic-resistant and hypervirulent can pose a serious threat to the healthcare system. Detection of virulence factors in KP isolates, especially combined with antibiotic resistance is very important because it allows for assessing the possible course and site of infection in the human body and is essential for development of treatment strategy.

## Materials and methods

### Bacterial isolates

A total of 109 KP isolates from human clinical materials such as swabs from wounds (13) and anus (17), urine (39) and blood samples (22), respiratory tract (16), and other samples (2) were used in this study (Table 7). The isolates were collected from 2018 to 2022 as part of routine diagnostic microbiology and obtained from various hospitalized patients in a district hospital in central Poland (Our Lady of Perpetual Help Hospital, Wołomin). Based on the Medical Microbiological Laboratory database, single KP isolates from different patients were used in the study. The patients were hospitalized in Nephrology, Internal, Surgery, Paediatric, Neurological, Intensive Care, Orthopaedics, Gynaecology units and Dialysis Station. The clinical materials were inoculated onto 5% sheep blood agar (bioMérieux, Marcy I’Etoile, France) and MacConkey agar (Oxoid, Basingstoke Hampshire, UK) and incubated for 24 h at 37 °C under aerobic conditions. Identification of bacterial isolates was carried out using colony morphology, Gram’s staining, and finally by VITEK 2 Compact System. The *K. pneumoniae* ATCC BAA-2473 reference strain was used as a control. For identification of bacteria by VITEK, Gram-negative ID cards (GN Reference 21 341) were used. Pf/Pr1 primers were used for KP identification as described earlier^53^.

**Table 7:** *K. pneumoniae* isolation sources.

Source of isolates	No. of isolates
Urine	39
Blood	22
Respiratory tract	16
Anus	17
Wound	13
Other	2

### Antimicrobial susceptibility testing

Antimicrobial susceptibility testing was performed using the disc-diffusion method on Mueller–Hinton agar (bioMérieux), using antibiotic discs (Oxoid, Basingstoke Hampshire, UK), according to the guidelines of the European Committee on Antimicrobial Susceptibility Testing (EUCAST 2020). The isolates were tested against penicillins: amoxicillin plus clavulanic acid (AMC, 20 µg + 10 µg), piperacillin plus tazobactam (TZP, 30 µg + 6 µg); cephalosporins: cefuroxime (CXM, 30 µg), cefotaxime (CTX, 5 µg); carbapenems: imipenem (IMP, 10 µg), meropenem (MEM, 10 µg); fluoroquinolones: ciprofloxacin (CIP, 5 µg); aminoglycosides: amikacin (AMK, 30 µg), gentamicin (GM, 10 µg); other drugs: trimethoprim plus sulfamethoxazole (SXT, 1.25 µg + 23.75 µg). Quality control was accessed using KP ATCC BAA-2473. Extended spectrum beta-lactamases (ESBLs) production was detected using the double disk synergy test^54^. The combined disc assay using imipenem and imipenem/ethylenediaminetetraacetate discs was used to confirm production of metallo-β-lactamases (MBLs) by isolates showing resistance to imipenem^55^. The RESIST-3 O.K.N. K-SeT rapid diagnostic test (Coris BioConcept, Gembloux, Belgium) for the detection in bacterial culture of carbapenemase class A (KPC), New Dehli metallo-β-lactamase (carbapenemase class B, NDM-1) and carbapenemase class D (OXA-48), was used. Isolates resistant to 3 or more classes of antimicrobial agents were considered MDR.

### DNA isolation

Genomic DNA from bacterial cells was isolated by using the NucleoSpin Microbial DNA (Macherey–Nagel GmbH&Co.KG, Düren, Germany) according to the manufacturer’s protocol.

### Primers and PCR conditions

The primer sequences specific for the identified genes, synthesized at Institute of Biochemistry and Biophysics, Polish Academy of Sciences (Warsaw, Poland), are listed in Table 8.

**Table 8:** Primers used for PCR.

Target gene	Primer sequences (5′ → 3′)	Length (bp)	Reference
Capsular type (primer)			
K1 (*magA*)	F: GGTGCTCTTTACATCATTGC	1283	Fang et al.^56^
	R: GCAATGGCCATTTGCGTTAG		
K2 (K2*wzy*)	F: GACCCGATATTCATACTTGACAGAG	641	Turton et al.^57^
	R: CCTGAAGTAAAATCGTAAATAGATGGC		
K5 (K5*wzx*)	F: TGGTAGTGATGCTCGCGA	280	Turton et al.^57^
	R: CCTGAACCCACCCCAATC		
K54 (*wzx*K54)	F: CATTAGCTCAGTGGTTGGCT	881	Fang et al.^58^
	R:GCTTGACAAACACCATAGCAG		
K57 (*wzy*K57)	F: CTCAGGGCTAGAAGTGTCAT	1037	Fang et al.^58^
	R: CACTAACCCAGAAAGTCGAG		
K20 (*wzy*K20)	F: CGGTGCTACAGTGCATCATT	741	Fang et al.^58^
	R: GTTATACGATGCTCAGTCGC		
*mrkD*	F: CCACCAACTATTCCCTCGAA	240	El Fertas-Aissani et al.^30^
	R: ATGGAACCCACATCGACATT		
*wcaG*	F: GGTTGGKTCAGCAATCGTA	169	Turton et al.^52^
	R: ACTATTCCGCCAACTTTTGC		
*rmpA*	F: ACTGGGCTACCTCTGCTTCA	516	Nadasy et al.^59^
	R: CTTGCATGAGCCATCTTTCA		
*fimH*	F: TGCTGCTGGGCTGGTCGATG	688	Yu et al.^60^
	R: GGGAGGGTGACGGTGACATC		
*entB*	F: ATTTCCTCAACTTCTGGGGC	371	El Fertas-Aissani et al.^30^
	R: AGCATCGGTGGCGGTGGTCA		
*irp-1*	F: TGAATCGCGGGTGTCTTATGC	238	El Fertas-Aissani et al.^30^
	R: TCCCTCAATAAAGCCCACGCT		
*iroD*	F: GCATAGGCGGATACGAACAT	531	Luo et al.^61^
	R: CACAGGGCAATTGCTTACCT		
*iroN*	F: CTGTCGGCATCGGTTTTATT	556	Yu et al.^60^
	R: TGGCGTGTCGATTATTACCA		
*clbA*	F: ATGAGGATTGATATATTAATTGGAC	735	Lai et al.^46^
	R: ATTCTGCCCATTTGACGAATG		
*clbB*	F: GATTTGGATACTGGCGATAACCG	732	Lai et al.^46^
	R: CCATTTCCCGTTTGAGCACAC		
*kfu*	F: ATAGTAGGCGAGCACCGAGA	520	Yu et al.^60^
	R: AGAACCTTCCTCGCTGAACA		
K. pneumoniae 16S-23S ITS*	Pf: ATTTGAAGAGGTTGCAAACGAT	130	Liu et al.^53^
	Pr1: TTCACTCTGAAGTTTTCTTGTGTTC		

*Internal transcribed spacer.

The simplex PCR for each gene was performed in a 25 µL volume containing 1 µL of DNA template, 12.5 µL of the PCR Mix Plus HGC (0.1 U/µL of Tag DNA polymerase, 4 mM of MgCl_2_, 0.5 mM of each dATP, dCTP, dGTP and dTTP) (A&A Biotechnology, Gdynia, Poland) and the specific primers at concentration of 200 nM.

The thermal cycling conditions included predenaturation at 95 °C for 4 min, 35 cycles of denaturation at 95 °C for 0.5 min, annealing of primers at 58 °C for 0.5 min and extension at 72 °C for 1 min. Amplification was carried out in the Eppendorf Mastercycler Nexus Gradient (Hamburg, Germany). A negative control was composed of all components of the PCR mixture, except for a template DNA. The positive controls included the genomic DNA from the isolates in which the presence of the searched genes was previously found in the genome. These controls were included in each test run. The PCR products were analysed by electrophoresis in 1.5% agarose gels stained with ethidium bromide. Molecular size markers (Sigma-Aldrich) were also run for verification of product size. The gel was electrophoresed in a 2 × Tris–borate buffer at 70 V for 1.5 h. The PCR amplicons were visualized using UV light (Syngen Imagine, Syngen Biotech, Wrocław, Poland)^62^.

### Data analyses

Chi-squared statistics in Statistix 11.0 (Analytical Software, Tallahassee, FL, USA) was used for testing significance of differences in the frequency of presence of resistance to antimicrobial agent or in the frequency of presence of gene between pairs of proportions (percentages) of KP isolates coming from various clinical materials. Differences at *p* ≤ 0.05 were regarded as significant, while at *p* ≤ 0.01 as highly significant.

### Ethical approval

According to Polish law and the regulations of the Scientific Research Ethics Committee established by the Rector’s Order No 128/2020, Siedlce University of Natural Sciences and Humanities, no ethical approval was required for this study and all the data were anonymous. No clinical samples were used in this study, we used clinical bacterial isolates that were obtained from the hospital.

## Data Availability

The datasets used and analysed during the current study are available from the corresponding author on reasonable request.
